# Gamma-aminobutyric acid associated research in Parkinson’s disease: an exploratory trends analysis

**DOI:** 10.3389/fnagi.2025.1655660

**Published:** 2026-01-27

**Authors:** Sheng-Qiang Zhou, Fang Liu, Yan-Jun Chen, Ming-Rong Xie

**Affiliations:** 1 National TCM Master Liu Zuyi Inheritance Studio, Hunan Provincial Hospital of Integrated Traditional Chinese and Western Medicine (The Affiliated Hospital of Hunan Academy of Traditional Chinese Medicine), Changsha, China; 2 Graduate School of Hunan University of Chinese Medicine, Changsha, China; 3 The First Clinical College of Nanjing University of Chinese Medicine, Nanjing, China

**Keywords:** basal ganglia, gamma-aminobutyric acid, oxidative stress, Parkinson’s disease, α-synuclein

## Abstract

**Background:**

Parkinson’s disease (PD) is a neurodegenerative disease characterized by the progressive loss of dopaminergic neurons. Gamma-aminobutyric acid (GABA), as a key inhibitory neurotransmitter, participates in physiological processes such as cognition and motor control by regulating the balance of neuronal excitability. Related studies have found that the GABAergic signaling abnormalities in the basal ganglia and thalamocortical circuits are closely associated with the motor dysfunction and non-motor symptoms of PD. This study aimed to analyze GABA and PD research literature to further identify hotspots, frontiers, and development directions.

**Method:**

Data were obtained from the Web of Science, Scopus, and PubMed databases. VOSviewer and CiteSpace were used to visualize and perform quantitative analyses.

**Results:**

From 2001 to 2024, a total of 630 publications related to GABA and PD were identified, and the annual publication count fluctuated with an overall upward trend. The United States and China contributed a large number of publications. Karolinska Institute was the leading research institution. *Neuroscience* published the most papers related to GABA and PD. Dr. Morari, Michele was the most prolific author. The keywords with high frequency focused on the core pathological mechanisms and the imbalance of neurotransmitters, the neural anatomical structure and functional circuits, the main research methods and models, and the treatment strategies. In recent years, α-synuclein, oxidative stress, and anxiety have emerged as research topics with higher burst intensity.

**Conclusion:**

This study delineates a comprehensive knowledge structure of GABA and PD research. Enhanced collaboration among authors across institutions and countries is pivotal to advancing the field. The mechanism of GABA in the basal ganglia region is a focus of current research. The detrimental effects of α-synuclein and oxidative stress on the GABAergic system and the non-motor symptom of anxiety are likely to be the frontiers of future research.

## 1 Introduction

Parkinson’s disease (PD) is a neurodegenerative disorder characterized by the degeneration of dopaminergic neurons and a significant reduction in dopamine (DA) levels (Kalia and Lang, 2015).

The motor symptoms of PD include resting tremor, rigidity, and postural instability, accompanied by non-motor symptoms such as depression, insomnia, and cognitive impairment (Bloem et al., 2021). Currently, although DA replacement therapy can alleviate some symptoms, it cannot halt disease progression. Consequently, exploring novel therapeutic targets has become a research focus.

Gamma-aminobutyric acid (GABA), a vital inhibitory neurotransmitter, maintains the excitation-inhibition balance within neural networks by activating ionotropic GABA_A_ receptors and metabotropic GABA_B_ receptors (Farrant and Nusser, 2005). Under physiological conditions, the GABAergic system plays a key role in regulating motor coordination, emotional stability, the sleep–wake cycle, and cognitive memory (Gerfen and Surmeier, 2011; Möhler, 2012; Saper et al., 2005; Buzsáki and Wang, 2012). Under pathological conditions, abnormal GABA signaling is closely associated with PD, epilepsy, Alzheimer’s disease, and depression (Wong et al., 2003; Shetty and Bates, 2016).

Related studies have shown that GABA is implicated in the pathogenesis and symptom progression of PD. Autopsy studies of PD patients revealed degeneration of GABAergic neurons in the basal ganglia, accompanied by markedly reduced GABA levels (Gerlach et al., 1996). PET imaging in early PD patients demonstrated GABAergic dysfunction in the putamen and cortical regions (Takashima et al., 2022). Degeneration of dopaminergic neurons in PD leads to an imbalance within the basal ganglia-thalamocortical circuit. Within this circuit, the GABAergic system modulates the overactivity of the indirect pathway in the basal ganglia, thereby suppressing excitatory output from the thalamus to the cortex. This suppression is thought to exacerbate bradykinesia and rigidity in PD (Obeso et al., 2008). Targeting GABA receptors or enhancing GABA synthesis may restore inhibitory tone and ameliorate motor symptoms. Investigating the relationship between GABA and PD could provide novel insights into the disease’s pathogenesis and the development of targeted therapeutic strategies, potentially delaying PD progression.

Bibliometrics, a discipline involving the quantitative analysis of academic literature using statistical methods, reveals the developmental trajectory, research hotspots, collaboration networks, and knowledge structure of a specific field by mining information such as keywords, authors, institutions, citation networks, and temporal trends within publication data. Applying bibliometrics to explore the relationship between GABA and PD can intuitively present research focus, identify research gaps, trace collaboration networks, and provide direction for interdisciplinary research.

## 2 Methods

### 2.1 Data search

**Figure 1:**
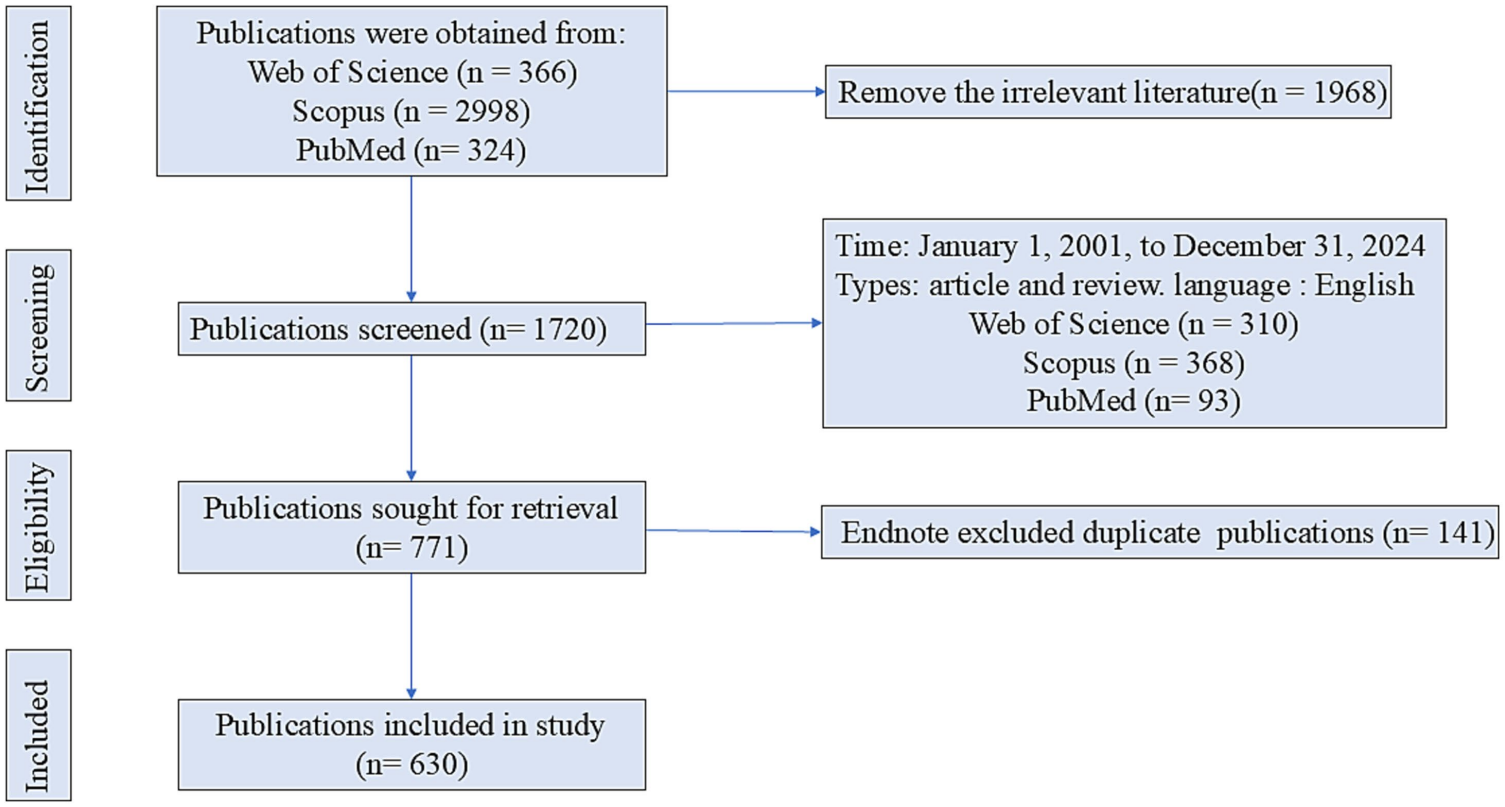
Literature search and selection flowchart.

Data were obtained from the Web of Science (WoS), Scopus, and PubMed databases. The search strategy employed was as follows: (i) WoS: ((TS = (gamma-aminobutyric acid)) OR TS = (4-Aminobutyric acid)) AND TS = (Parkinson’s disease). (ii) Scopus: (([TITLE-ABS-KEY](gamma-aminobutyric acid)) OR ([TITLE-ABS-KEY]((4-Aminobutyric acid))) AND ([TITLE-ABS-KEY](Parkinson’s disease)). (iii) PubMed: ((gamma-aminobutyric acid[Title/Abstract]) OR (4-Aminobutyric acid[Title/Abstract])) AND (Parkinson’s disease[Title/Abstract]). The inclusion criteria were as follows: (i) Publication date from January 1, 2001, to December 31, 2024; (ii) Publication type restricted to articles and reviews; (iii) Language restricted to English. The exclusion criteria were: (i) The EndNote software eliminated duplicate publications by the same author. (ii) Exclusion of publications irrelevant to the research topic. Following the application of these criteria, 630 publications relevant to GABA and PD were identified (Figure 1).

### 2.2 Data analysis

We employed the software tools VOSviewer (1.6.18) and CiteSpace (6.2.R3) to analyze annual publication trends, countries, institutions, journals, authors, and keywords. This approach is consistent with methodologies established in prior literature (Chen et al., 2025). CiteSpace is a specialized tool for visualizing and mapping knowledge domains within scientific literature. It facilitates the identification of emerging research frontiers, evolutionary trends, and the dynamic patterns of disciplinary development (Chen, 2004). VOSviewer is widely recognized for its intuitive visualization of co-occurrence networks. It utilizes color clustering and density views to illustrate the association strength among elements such as keywords and authors, enabling the efficient construction of data networks (van Eck and Waltman, 2010).

## 3 Results

### 3.1 Publication trends

**Figure 2:**
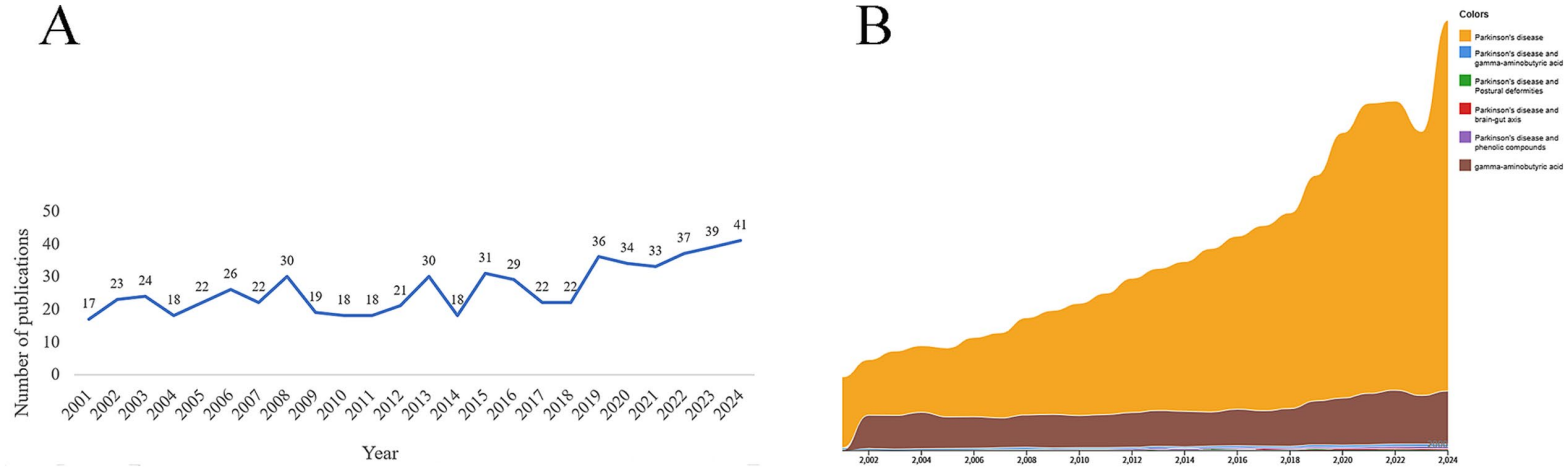
Analysis of publication trends. **(A)** Annual number of publications on GABA and PD. **(B)** Proportion of research topics in PD publications.

A total of 630 publications related to GABA and PD were obtained. Annual publication numbers exhibited an upward fluctuation (Figure 2A). Research on GABA in PD accounted for a relatively small proportion of all published PD research (Figure 2B). The research on GABA in PD accounted for a relatively small proportion of the PD-related studies listed in the WoS (95,413 publications) (Figure 2B).

### 3.2 Country distribution

**Figure 3:**
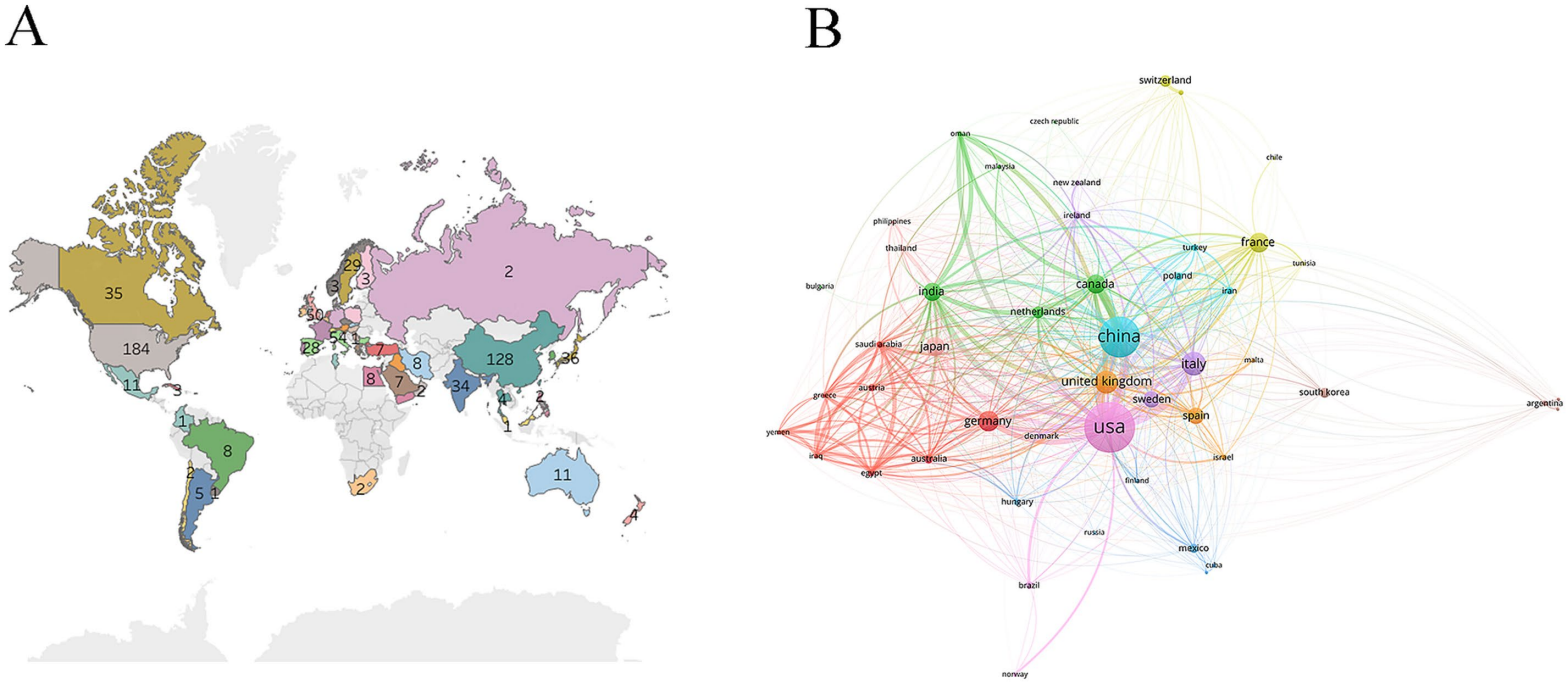
Country analysis. **(A)** Geographic distribution of publications. **(B)** International collaborative network map. The size of the nodes represents the number of publications from each country, and the lines represent the collaboration between countries.

The world map indicated that GABA-PD publications were concentrated in Asia and North America, with relatively few publications from South America and Africa (Figure 3A). In the country collaboration network, node size corresponds to publication volume, and line thickness indicates collaboration strength (Figure 3B). The United States and China have made significant contributions to GABA-PD research. The United States was the most active country (184 publications), followed by China (128 publications) and Italy (54 publications) (Table 1).

**Table 1:** The top 10 countries.

Rank	Country	Number of publications	Citations
1	USA	184	4,152
2	China	128	1,475
3	Italy	54	769
4	United Kingdom	50	1764
5	Germany	41	1,029
6	France	39	699
7	Japan	36	663
8	Canada	35	1,376
9	India	34	498
10	Sweden	29	215

### 3.3 Research institutions

Karolinska Institute was the most active institution (21 publications), followed by the University of Ferrara (16 publications) and Emory University (14 publications). The top 10 institutions represented three continents: North America, Europe, and Asia, encompassing six countries (Table 2).

**Table 2:** The top nine institutions.

Rank	Institution	Documents
1	Karolinska Institute	21
2	University of Ferrara	16
3	Emory University	14
4(tie)	Xi’an Jiaotong University	12
4(tie)	University of Toronto	12
6(tie)	Qingdao University	5
6(tie)	Shandong University	5
6(tie)	Tianjin University	5
6(tie)	University of Manchester	5

### 3.4 Journals

**Figure 4:**
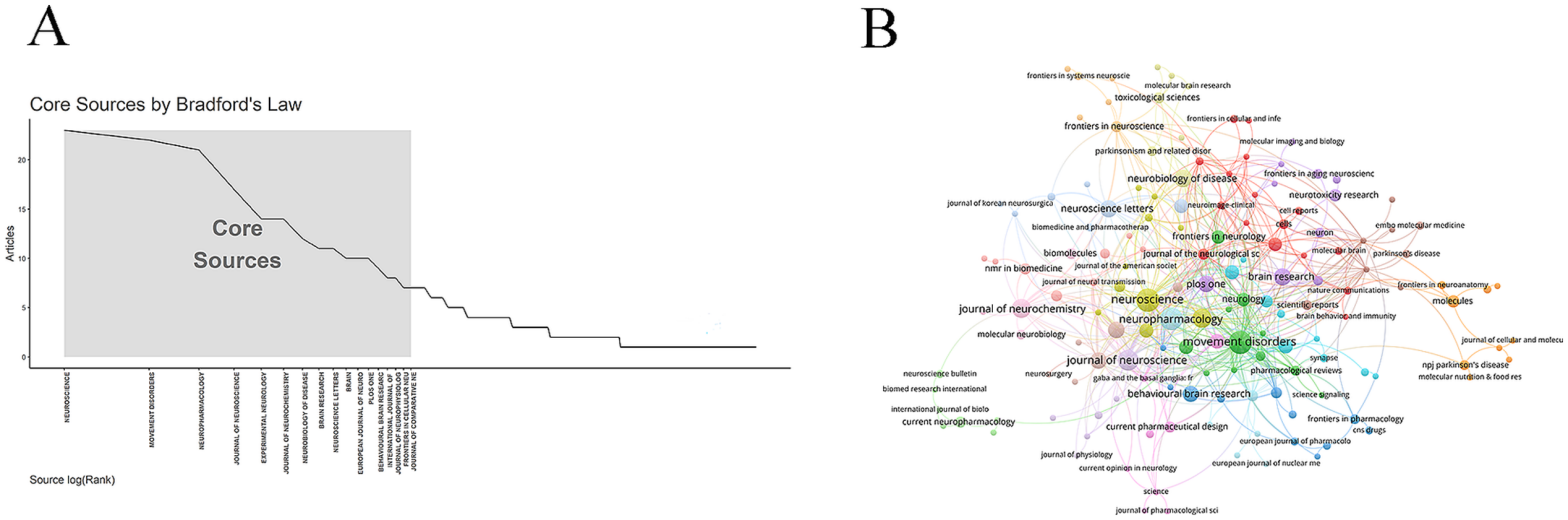
Journal analysis. **(A)** Core journal. **(B)** Journal network diagram.

According to Bradford’s law, we identified 17 core journals relevant to the topic (Figure 4A). Within PD research, studies on GABA have not yet achieved wide interdisciplinary integration; the field remains predominantly centered within neurology. *Neuroscience* published the most articles (23 publications), followed by *Movement Disorders* (22 publications) and *Neuropharmacology* (21 publications) (Figure 4B and Table 3).

**Table 3:** The top 10 journals.

Rank	Source	Documents	Citations	Average number of citations	IF	JCR
1	Neuroscience	23	371	16.13	2.9	Q2
2	Movement Disorders	22	356	16.18	7.4	Q1
3	Neuropharmacology	21	202	9.62	4.6	Q1
4	Journal of Neuroscience	17	391	23.00	4.4	Q1
5	Experimental Neurology	14	168	12.00	4.6	Q1
5	Journal of Neurochemistry	14	125	8.93	4.2	Q2
7	Neurobiology of Disease	12	332	27.67	5.1	Q1
8	Brain Research	11	46	4.18	2.7	Q3
8	Neuroscience Letters	11	207	18.82	2.5	Q3
10	Brain	10	438	43.80	11.7	Q1
10	PLoS One	10	246	24.60	2.9	Q2
10	European Journal of Neuroscience	10	343	34.30	2.7	Q3

IF, Impact Factor; JCR, Journal Citation Reports.

### 3.5 Authors

**Figure 5:**
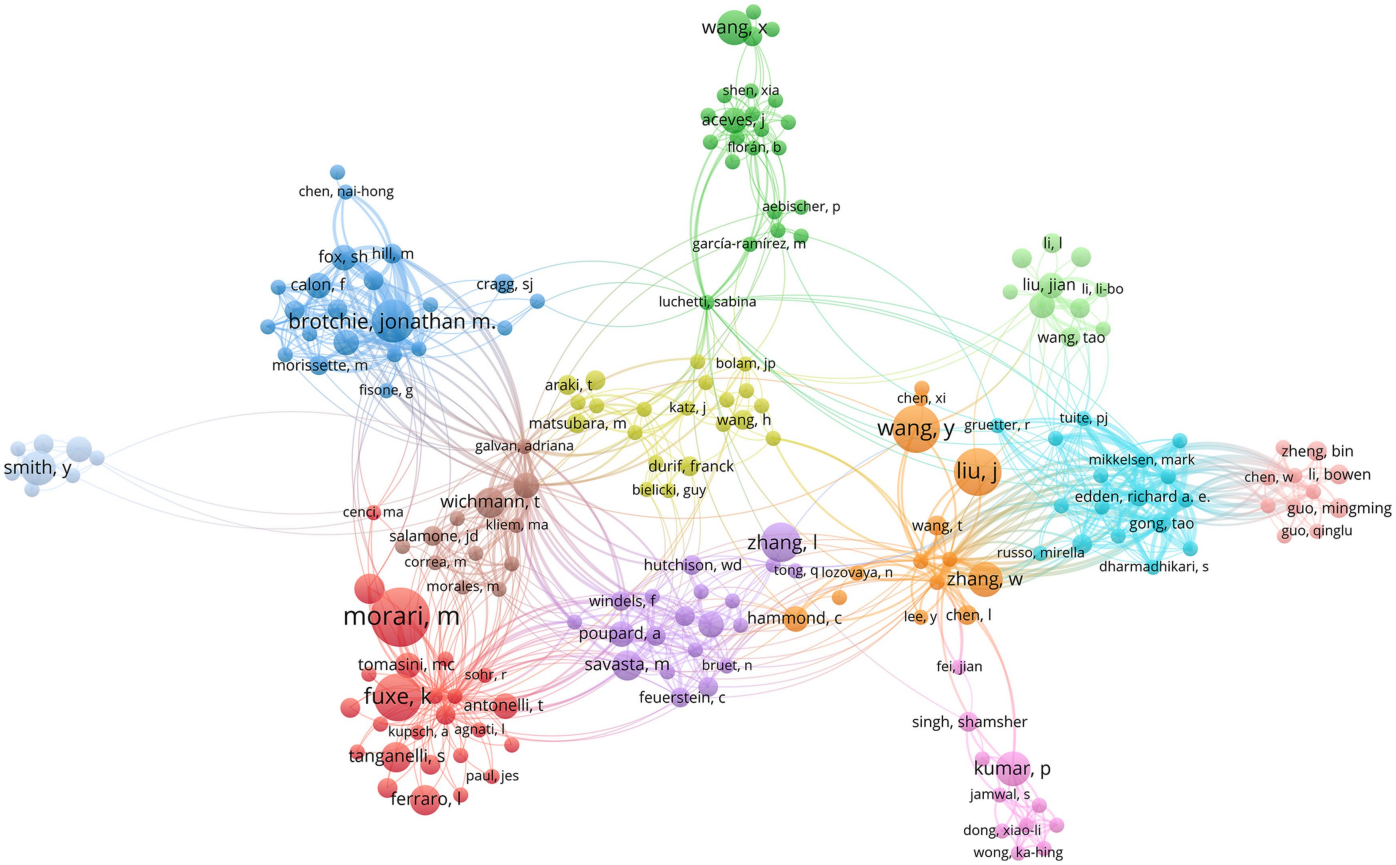
Author collaboration network.

Highly productive authors constitute a dominant force in this research field. In the author collaboration network, node size corresponds to publication volume (Figure 5). Dr. Morari, Michele was the most productive author (13 publications), followed by Dr. Jian Liu (12 publications) and Dr. Kjell Fuxe (10 publications) (Table 4). These prolific authors have contributed significantly to the advancement of the field.

**Table 4:** The top 10 authors.

Rank	Author	Documents	Country	Institution
1	Dr. Morari, Michele	13	Italy	University of Ferrara
2	Dr. Jian, Liu	12	China	Xi’an Jiaotong University
3	Dr. Fuxe, Kjell	10	Italy	University of Ferrara
4	Dr. Wichmann, Thomas	9	USA	Emory University
5	Dr. Brotchie, Jonathan M.	8	Canada	Toronto Western Hospital
5	Dr. Smith, Yoland	8	USA	Emory University
5	Dr. Savasta, Marc	8	France	Inserm
8	Dr. Kumar, Puneet	7	India	Maharaja Ranjit Singh Punjab Technical University
8	Dr. Di Paolo, Therese	7	Canada	Laval University
8	Dr. Singh, Shamsher	7	India	ISF College of Pharmacy

### 3.6 Keywords

**Figure 6:**
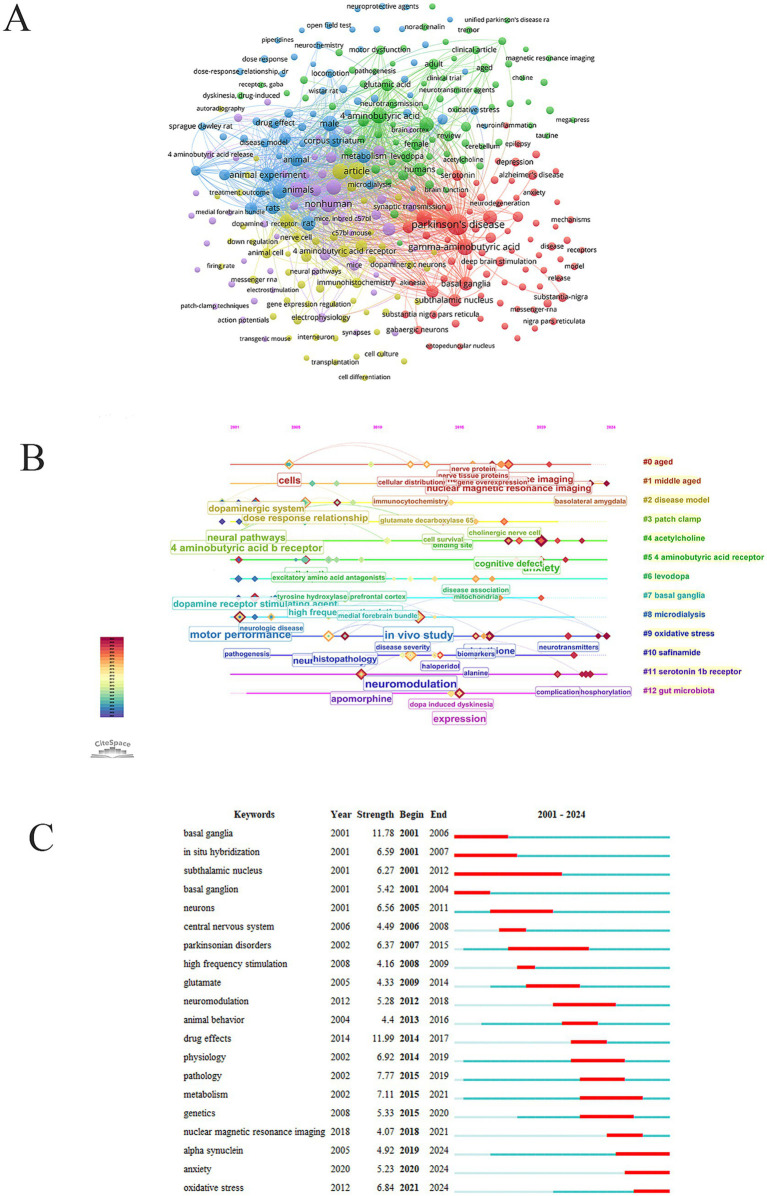
Keywords analysis. **(A)** Keywords network diagram. **(B)** Keywords cluster analysis. **(C)** Keywords with the strongest citation bursts.

High-frequency keywords reflect current research focus and enable researchers to rapidly identify core topics. Besides GABA (540 times) and PD (475 times), high-frequency keywords included animals (186 times), male (179 times), DA (174 times), glutamate (140 times), basal ganglia (131 times), metabolism (127 times), substantia nigra (98 times), subthalamic nucleus (92 times), neurons (92 times), globus pallidus (71 times), deep brain stimulation (46 times) (Figure 6A). Cluster #0 aged, #1: middle-aged, #2: disease model, #7: basal ganglia, #9: oxidative stress reflected the core background and pathophysiology of PD. Cluster #3: patch clamp, #4: acetylcholine, #5: 4-aminobutyric acid receptor, #8: microdialysis, #11: serotonin 1b receptor reflected the detailed research methods and complex neurotransmitter interactions. Cluster #6: levodopa, #10: safinamide, #12: gut microbiota reflected the transition from traditional treatment to cutting-edge exploration in PD (Figure 6B). Keyword burst refers to the sudden and significant increase in a keyword at a specific time. “α-synuclein” (2019–2024, strength = 4.92), “anxiety” (2020–2024, strength = 5.23), and “oxidative stress” (2021–2024, strength = 6.84) have exhibited high burst intensity in recent GABA-PD research (Figure 6C and Table 5).

**Table 5:** Burst keywords with example citations in recent years.

Burst keywords	Citations
	1. Cellular milieu imparts distinct pathological α-synuclein strains in α-synucleinopathies
α-synuclein	2. Trial of prasinezumab in early-stage Parkinson’s disease
	3. Discriminating α-synuclein strains in Parkinson’s disease and multiple system atrophy
	1. Blockade of pre- and post-synaptic GABAB receptors in the anteroventral bed nucleus of stria terminalis produces anxiolytic-like and anxiety-like effects in parkinsonian rats, respectively
anxiety	2. Nuclear localization of alpha-synuclein induces anxiety-like behavior in mice by decreasing hippocampal neurogenesis and pathologically affecting amygdala circuits
	3. Blockade of pre-synaptic and post-synaptic GABAB receptors in the lateral habenula produces different effects on anxiety-like behaviors in 6-hydroxydopamine hemiparkinsonian rats
	1. Role of GABA pathway in motor and non-motor symptoms in Parkinson’s disease: a bidirectional circuit
oxidative stress	2. L-Theanine ameliorated rotenone-induced parkinsonism-like symptoms in rats
	3. Inhibition of NADPH oxidase within midbrain periaqueductal gray decreases pain sensitivity in Parkinson’s disease via GABAergic signaling pathway

## 4 Discussion

### 4.1 General information

From 2001 to 2024, a total of 630 publications related to GABA and PD were identified. The annual publication count exhibited an upward trend with fluctuations. The United States and China have allocated substantial funding to neuroscience and Parkinson’s disease research, which has directly facilitated investigations into the GABAergic system. As leading contributors in the field, these nations frequently serve as central hubs in large-scale international collaborative networks, thereby reinforcing their prominent roles. Regarding global cooperation, the successful model of the Parkinson’s Progression Markers Initiative could be leveraged to establish an international “GABA-PD Alliance.” Such an alliance could enable the sharing of patient biospecimen repositories with GABA-related pathological data and support multicenter, longitudinal clinical studies to elucidate dynamic changes in the GABAergic system throughout PD progression. Karolinska Institute was the leading research institution. *Neuroscience* published the most papers on GABA and PD. Dr. Michele Morari was the most prolific author.

### 4.2 Hotspots and frontiers

High-frequency keywords directly reflect topics of widespread concern within a field and reveal current research focus. In addition to GABA and PD, high-frequency keywords included animals, male, DA, glutamate, basal ganglia, metabolism, substantia nigra (SN), subthalamic nucleus, neurons, globus pallidus (GP), and deep brain stimulation (DBS). The basal ganglia constitute a complex neural network responsible for motor control, learning, emotion regulation, and other functions. Its core nuclei include the striatum, GP, SN, and STN. These nuclei interact through both direct (promoting movement) and indirect (inhibiting movement) pathways to maintain motor balance (Yelnik, 2002). The core pathology of PD involves the degeneration of dopaminergic neurons in the SN pars compacta and reduced DA levels, leading to an imbalance between the indirect and direct pathways of the basal ganglia. Excessive activity within the indirect pathway, primarily mediated by GABAergic neurons, is an important mechanism underlying the development of motor symptoms in PD (Obeso et al., 2008). Glutamate, as an excitatory neurotransmitter, participates in PD progression (Blandini et al., 1996). In PD, decreased GABAergic inhibition and increased glutamatergic excitation form a vicious cycle, resulting in excessive inhibition of the basal ganglia output nuclei (GPi/SNr) on the thalamus and consequent movement disorders (Windels et al., 2003). DBS is an established clinical treatment for PD, and its mechanism of action may involve modulation of the STN-GPe network (Noor et al., 2024).

Keywords such as α-synuclein oxidative stress and anxiety have shown a high burst intensity in recent GABA and PD research. This trend highlights the most active and highly focused core directions and emerging focus in current PD research. α-Synuclein is the core pathological hallmark of PD; its aggregation propagation and toxicity are central to PD pathogenesis (Yaribash et al., 2025). Oxidative stress results from an imbalance between the production and clearance of reactive oxygen species within cells and is considered a key driver of neuronal death in PD (Jenner, 2003). Anxiety is a common non-motor symptom in PD patients and its pathophysiological mechanisms are closely linked to neurotransmitter dysregulation and neural circuit dysfunction associated with PD (Walsh and Bennett, 2001). Abnormal aggregation and propagation of α-synuclein can directly or indirectly induce oxidative stress which in turn exacerbates α-synuclein aggregation and toxicity forming a vicious cycle. The core pathological process of α-synuclein and oxidative stress extensively damages brain neurons and neural circuits (Surmeier et al., 2017). The basal ganglia-thalamocortical circuit and GABAergic interneurons and projection neurons within the limbic system are particularly vulnerable to this damage. Dysfunction of GABAergic signaling in these circuits is regarded as a key mechanism contributing to neuropsychiatric symptoms such as anxiety in PD (Li et al., 2024). These foci reflect an in-depth exploration of PD’S pathological mechanisms. The attention paid to anxiety as a non-motor symptom indicates that it has become a clinically significant research direction in the field of PD studies. The GABAergic system plays a pivotal role in this context acting as a key link connecting upstream core pathology with downstream key clinical symptoms.

### 4.3 Clinical research

In the evaluation of clinical trials targeting the GABAergic system for PD interventions, studies vary in efficacy, methodology, and clinical translational potential. A clinical trial of nabilone (*n* = 7) showed that it significantly alleviated levodopa-induced dyskinesia. The mechanism may involve inhibition of GABA reuptake in the GP and enhancement of inhibitory transmission; however, this trial had a small sample size, did not assess core motor symptoms, and lacked long-term safety data (Sieradzan et al., 2001). A study on the GABA_A_ receptor antagonist flumazenil (*n* = 16) reported improved motor flexibility, although without significant improvement in UPDRS-III scores. Limitations included the short duration of the single-dose effect, lack of long-term safety data, and the common adverse drug reaction of dizziness (Ondo and Silay, 2006). The Zonisamide study (*n* = 389) demonstrated that 50 mg/day significantly reduced “off” time without increasing the risk of dyskinesia or hallucinations. However, limitations remain: Zonisamide’s multi-target mechanism (MAO-B inhibition, calcium channel regulation) makes it difficult to attribute effects solely to a single GABAergic pathway, and the inclusion of patients already receiving combined treatments such as dopamine agonists may have weakened the observed effect (Murata et al., 2015). A 4-month randomized controlled trial of the NKCC1 inhibitor bumetanide (*n* = 44) showed that improvement in UPDRS-III scores during the OFF period did not differ significantly between the bumetanide and placebo groups. Furthermore, tolerance was poor, possibly due to insufficient brain penetration of the drug and systemic side effects (Damier et al., 2024). A randomized, double-blind trial of probiotic-M8 combined with conventional drugs for PD treatment (*n* = 82) reported significant synergistic benefits in improving non-motor symptoms and some motor functions (UPDRS-III score), with good safety. However, limitations include a small sample size, high dropout rate, inadequate analysis of the primary motor endpoint, and methodological constraints resulting in a relatively low level of evidence (Sun et al., 2022). A clinical trial investigating DBS for PD provided direct neurochemical evidence for the role of the GP in human memory processing. The findings revealed the complex and specific regulatory patterns of basal ganglia circuits across different cognitive functions. In the enrolled PD patients, changes in GABA concentration within the GP were closely associated with the type of memory task performed: GABA levels increased during implicit memory tasks but decreased during explicit memory tasks (Buchanan et al., 2015). However, this study had several limitations, including a small sample size (*n* = 2) and the constrained temporal resolution of microdialysis technology. In a study conducted on PD patients undergoing STN-DBS treatment, brain chemical measurements revealed a significant decrease in GABA concentration in the thalamus (Stefani et al., 2011). The decrease in GABA concentration reduces the inhibitory effect on the thalamus, and is considered a key event for restoring motor function and achieving clinical efficacy. Overall, current clinical evidence for GABA-targeted therapy is generally of low quality (small sample sizes, short study durations). Strategies directly targeting GABA receptors or transporters have demonstrated limited efficacy and are associated with tolerance issues. In contrast, approaches indirectly modulating the GABAergic pathway (such as probiotics regulating neurotransmitter metabolism via the microbiota-gut-brain axis) show potential for improving non-motor symptoms, but alleviation of core motor deficits still requires optimization. Future research should integrate large-sample RCTs, targeted drug delivery technologies, and multi-omics mechanistic studies to clarify the precise therapeutic role of GABAergic modulation in PD.

### 4.4 Correlation between GABA and PD

GABAergic dysfunction contributes to motor symptoms in PD primarily through an imbalance in the basal ganglia’s direct/indirect pathways, leading to excessive inhibition of GPi/SNr output (DeLong and Wichmann, 2007). The successful application of GPi-targeted DBS is the most direct therapeutic manifestation of this mechanism (Wichmann and DeLong, 2016). The role of GABAergic interneurons within cortical-basal ganglia-thalamus circuit microcircuits, particularly in the generation of pathological oscillations, is under active investigation. Beyond the main output nuclei, numerous GABAergic interneurons are present within basal ganglia circuits (such as striatum, GPe, and STN) as well as in related cortical and thalamic regions. These interneurons play a crucial role in fine-tuning information flow and synchronizing neuronal activities, such as the generation of β oscillations in local microcircuits (Devergnas et al., 2014). In PD, DA deficiency may disrupt the normal function of these interneurons, leading to local network excitation-inhibition imbalance and pathological oscillations, which are closely associated with bradykinesia and rigidity (Gittis et al., 2011). The specific mechanisms of the GABA system in non-motor symptoms of PD (such as depression, anxiety, cognitive impairment, sleep disorders, and comorbidities) remain insufficiently studied. GABA, as the primary inhibitory neurotransmitter in the central nervous system, is widely distributed in brain regions involved in emotion, cognition, and sleep regulation (Luscher et al., 2011; Winsky-Sommerer, 2009). GABAergic dysfunction (such as loss of GABAergic neurons, altered receptor expression, and abnormal signal transduction) may significantly contribute to these non-motor symptoms. An in-depth exploration of how GABAergic changes in the cortex, limbic system, and brainstem contribute to specific non-motor symptoms is crucial for developing novel therapies targeting these aspects.

### 4.5 Challenge and opportunity

In GABA and PD research, challenges and opportunities coexist. GABAergic neurons in the basal ganglia are distributed across multiple nuclei, and their complex interactions complicate the precise localization of functional abnormalities. DA depletion leads to overactivity of the indirect pathway, which may exacerbate motor inhibition via GABAergic neurons; however, the specific mechanisms are not fully understood. Furthermore, animal models may not fully recapitulate changes in the human GABA system. Poor blood–brain barrier penetration, receptor tolerance, and long-term side effects limit the clinical application of GABAergic drugs. Future validation of drug safety and efficacy requires large-scale, multi-center clinical trials. Despite the complexity of the GABA system in PD posing research challenges, emerging technologies offer broad opportunities for precision therapy development. Gene-editing technologies or viral vectors could modulate GABAergic transmission and potentially repair aberrant circuits. Combining imaging (such as PET/fMRI) or molecular markers to identify PD patient subgroups could facilitate personalized treatment.

### 4.6 Limitations

In this study, only English-language publications were included during the literature search and screening process. Although this approach is common in bibliometric research to ensure data source consistency and reproducibility, it inevitably introduces a certain degree of language bias. The excluded non-English literature accounted for 8.21% of the retrieved records. Consequently, the findings and analyses presented herein primarily reflect the research landscape of GABA in PD from the perspective of international English-language journals and may not fully represent the global situation in this field. Future studies could provide a more globally representative analysis by incorporating literature from multilingual databases.

## 5 Conclusion

This study provides a visual analysis of the role of GABA in PD research. Strengthening collaboration among institutions and countries will help advance this field. Research primarily focuses on the mechanisms and interventions related to neural circuit dysfunction in PD. α-synuclein, oxidative stress, and anxiety are likely to represent key research directions in the coming years.

## Data Availability

The original contributions presented in the study are included in the article/supplementary material, further inquiries can be directed to the corresponding authors.
